# Central Serous Chorioretinopathy in Kidney Transplant Recipients: A Report of Three Cases

**DOI:** 10.7759/cureus.110945

**Published:** 2026-06-16

**Authors:** Salima Serroukh, Abdallah Elhassan, Qods Yacoubi, Loubna Benamar, Naima Ouzeddoun

**Affiliations:** 1 Department of Nephrology, Dialysis and Kidney Transplantation, Ibn Sina University Hospital Centre, Rabat, MAR; 2 Department of Ophthalmology B, Rabat Specialty Hospital, Ibn Sina University Hospital Center, Rabat, MAR

**Keywords:** central serous chorioretinopathy, corticosteroids, immunosuppressive therapy, kidney transplantation, ophthalmological complications

## Abstract

Central serous chorioretinopathy (CSCR) is a retinal disorder characterized by subretinal fluid accumulation in the macular region and is commonly associated with psychological stress and prolonged corticosteroid exposure. In kidney transplant recipients, CSCR represents an underrecognized complication related to immunosuppressive therapy. We report three cases of CSCR occurring in male kidney transplant recipients receiving corticosteroid-based immunosuppression. Clinical presentation was dominated by decreased visual acuity and blurred vision. Diagnosis was established by ophthalmologic examination and fluorescein angiography in all cases, with optical coherence tomography (OCT) findings available in two patients. Two patients, aged 35 and 37 years, developed CSCR within weeks following transplantation while receiving corticosteroids, mycophenolate mofetil, and calcineurin inhibitors. In the third case, a 65-year-old diabetic patient was diagnosed incidentally during routine ophthalmologic follow-up. Gradual tapering of corticosteroids resulted in favorable clinical and visual outcomes in all patients. CSCR in kidney transplant recipients is mainly associated with prolonged corticosteroid therapy and may occur from the early post-transplant period to several years after transplantation. Early diagnosis and close collaboration between nephrologists and ophthalmologists are essential to optimize management, prevent visual complications, and preserve patients' quality of life.

## Introduction

Central serous chorioretinopathy (CSCR) is a retinal disorder characterized by the accumulation of subretinal fluid in the macular region. It results from abnormalities in choroidal circulation and dysfunction of the retinal pigment epithelium (RPE), leading to variable degrees of visual impairment [1]. CSCR is commonly associated with several risk factors, including psychological stress, endogenous or exogenous corticosteroid exposure, and other conditions affecting choroidal vascular permeability [2].

In kidney transplant recipients, prolonged or repeated corticosteroid use as part of immunosuppressive therapy represents a major risk factor for the development of CSCR. This ophthalmologic complication is of particular concern in this immunocompromised population because of its potential impact on visual function and quality of life [3,4].

Management of CSCR in this setting requires a careful balance between maintaining adequate immunosuppression to prevent graft rejection and addressing visual complications. Several studies suggest that cautious corticosteroid tapering may improve CSCR without significantly increasing the risk of rejection. However, this approach requires close ophthalmologic monitoring and strong collaboration between nephrologists and ophthalmologists [5].

In this case series, we report three cases of CSCR occurring in kidney transplant recipients receiving corticosteroid therapy among a cohort of 176 patients followed at Ibn Sina University Hospital Centre, Rabat, Morocco. We aimed to describe their clinical presentation, angiographic findings, and outcomes.

## Case presentation

Case 1

A 35-year-old man underwent kidney transplantation from a living-related donor after one year of hemodialysis. The underlying nephropathy was of unknown etiology. Immunosuppressive therapy included thymoglobulin, corticosteroids, mycophenolate mofetil (MMF), and tacrolimus.

Three weeks after transplantation, he developed acute cellular rejection, which was treated with IV methylprednisolone pulses at 15 mg/kg/day for 3 days.

**Figure 1:**
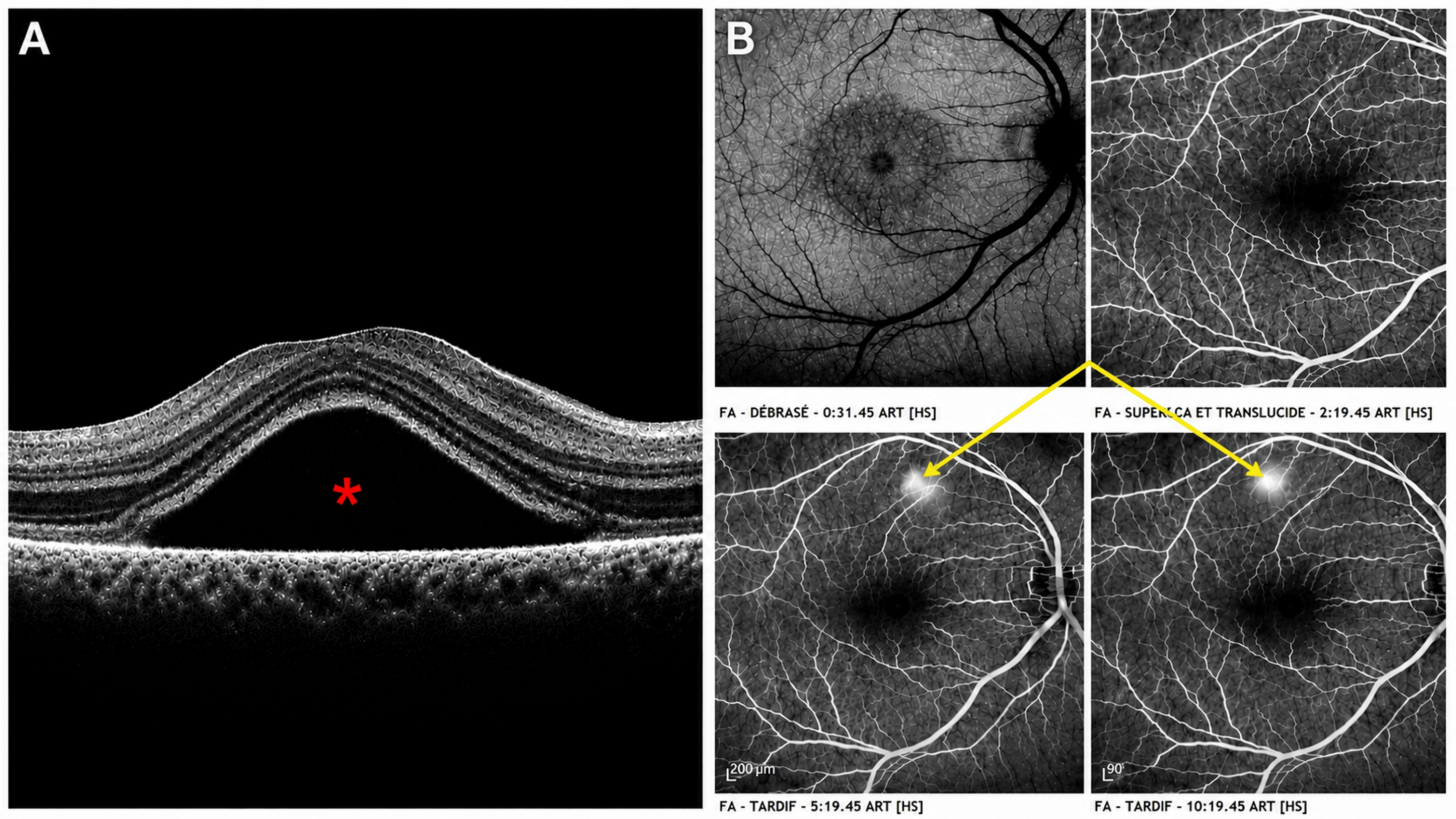
(A) Macular OCT demonstrating subretinal fluid accumulation in the subfoveal space, with elevation of the neurosensory retina, loss of the normal foveal depression, and preservation of the Bruch’s membrane-retinal pigment epithelium complex (*), resulting in serous neurosensory retinal detachment. No significant disruption of the outer retinal layers or other signs of chronicity are observed. (B) Fluorescein angiography showing a leakage point along the superotemporal vascular arcade (yellow arrows). OCT: Optical coherence tomography.

Six weeks post-transplantation, he presented with a sudden decrease in visual acuity in the right eye. Fundoscopic examination revealed serous retinal detachment. Optical coherence tomography (OCT) demonstrated subretinal fluid with elevation of the neurosensory retina and preservation of the hyperreflective Bruch’s membrane-RPE complex. No evidence of secondary choroidal neovascularization was identified. Fluorescein angiography showed a leakage point along the superotemporal vascular arcade (Figure 1).

CSCR was diagnosed, and corticosteroids were progressively tapered. The clinical course was favorable, with complete resolution of symptoms after six months.

Case 2 

A 37-year-old man underwent kidney transplantation from a living-related donor after one year of hemodialysis. The underlying nephropathy was unknown. Immunosuppressive therapy included thymoglobulin, corticosteroids, MMF, and cyclosporine.

**Figure 2:**
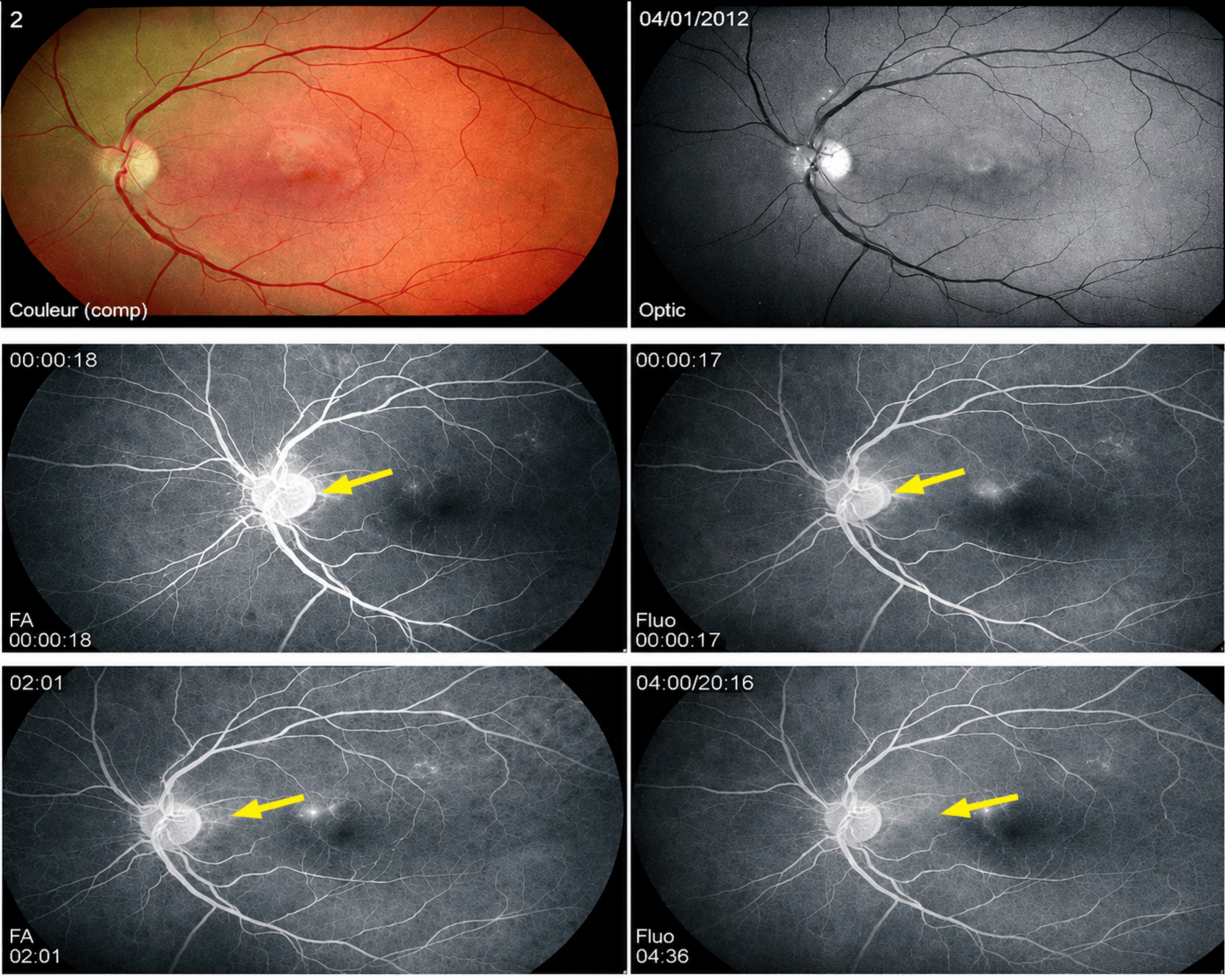
Fluorescein angiography demonstrating a focal leakage point (yellow arrows).

One month after transplantation, he presented with sudden visual acuity loss in the right eye associated with blurred vision. Fundoscopic examination revealed serous retinal detachment, which was confirmed by fluorescein angiography showing a focal leakage point (Figure 2).

CSCR was diagnosed, and corticosteroid therapy was gradually reduced. Visual symptoms resolved completely after four months.

Case 3

A 65-year-old man with end-stage kidney disease of unknown etiology underwent kidney transplantation from a living unrelated donor after two years of hemodialysis, without induction therapy. Maintenance immunosuppression included corticosteroids, MMF, and cyclosporine.

Three years after transplantation, he developed hypertension, overweight, and post-transplant diabetes mellitus, leading to corticosteroid discontinuation. Seventeen years post-transplantation, following an episode of rejection, he was treated with methylprednisolone pulses followed by oral corticosteroids and rituximab.

Three years later, he was admitted for progressive graft dysfunction with proteinuria and donor-specific antibodies. Biopsy confirmed chronic rejection, leading to another course of corticosteroid pulses.

**Figure 3:**
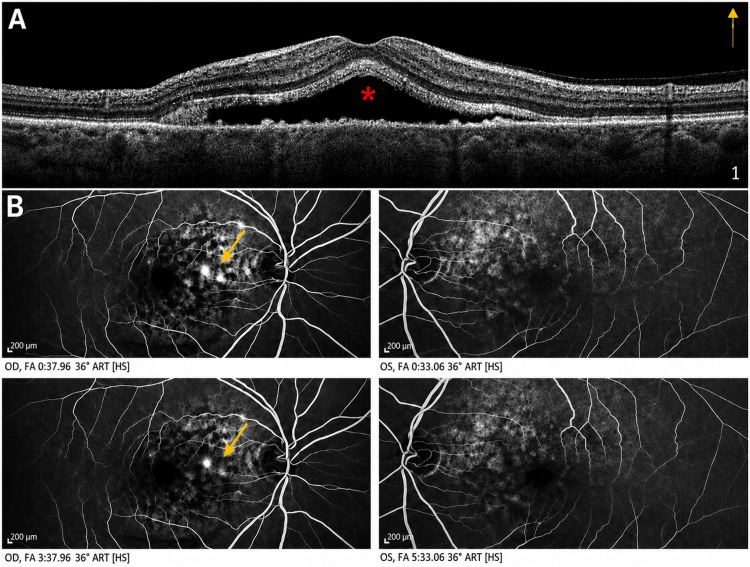
(A) Macular OCT demonstrating extensive subretinal fluid accumulation with a large serous neurosensory retinal detachment and irregularity of the Bruch’s membrane-retinal pigment epithelium complex (*). No intraretinal fluid or macular edema is identified. (B) Fluorescein angiography showing multiple hyperfluorescent focal leakage sites in the interpapillomacular region associated with diffuse retinal pigment epithelium alterations, consistent with chronic central serous chorioretinopathy (yellow arrows). OCT: Optical coherence tomography.

Twenty years after kidney transplantation, CSCR was incidentally diagnosed during routine ophthalmologic screening for diabetic retinopathy in an otherwise asymptomatic patient. OCT demonstrated a large area of subretinal fluid with serous neurosensory retinal detachment and irregularity of the Bruch’s membrane-RPE complex. No macular edema or evidence of secondary choroidal neovascularization was identified. Fluorescein angiography revealed multiple hyperfluorescent focal leakage sites in the interpapillomacular area associated with diffuse RPE alterations, findings consistent with chronic CSCR (Figure 3).

Progressive tapering of corticosteroid therapy resulted in significant radiologic improvement after one month of follow-up.

## Discussion

Central serous chorioretinopathy (CSCR) is an uncommon but clinically significant ophthalmologic complication in kidney transplant recipients. Its reported prevalence ranges from 1% to 6%, although this varies across studies and follow-up duration [3,4]. Importantly, CSCR may occur at any time after transplantation, from the early postoperative period to more than two decades later, highlighting the need for long-term and regular ophthalmologic surveillance, even in asymptomatic patients [6,7].

The association between CSCR and immunosuppressive therapy, particularly corticosteroids, is well established. Several case series and retrospective studies involving kidney and other solid organ transplant recipients have consistently identified corticosteroid exposure as the primary risk factor [5,8]. The underlying pathophysiology is multifactorial and involves increased choroidal vascular permeability, disruption of the blood-retinal barrier, and dysfunction of the RPE, leading to subretinal fluid accumulation and neurosensory retinal detachment [1,6,9,10].

CSCR most frequently develops during periods of high corticosteroid exposure, such as the early post-transplant phase or during acute rejection episodes, as illustrated in our Cases 1 and 2 and consistently reported in transplant cohorts [5,7,8]. Clinically, the presentation ranges from sudden visual acuity loss to blurred vision, dyschromatopsia, and metamorphopsia. Fluorescein angiography remains a reference imaging modality for identifying leakage points and confirming the diagnosis, while OCT allows quantification of subretinal fluid and choroidal thickness, providing essential information for monitoring and therapeutic decision-making. These findings are consistent with previously published data in kidney and other solid organ transplant recipients [7,8].

According to both the literature and our findings, gradual corticosteroid tapering, when clinically feasible, remains the cornerstone of CSCR management and is frequently associated with resolution of subretinal fluid without compromising graft function [5]. In our series, corticosteroid reduction was individualized according to each patient's immunological risk profile and allograft status. Two patients developed CSCR following exposure to high-dose corticosteroid therapy administered for rejection episodes. One patient developed CSCR after treatment of acute cellular rejection with IV methylprednisolone pulses at 15 mg/kg/day for three days, followed by oral prednisone at 1 mg/kg/day, approximately 60 mg/day, whereas another developed the condition after repeated courses of high-dose corticosteroids administered for chronic antibody-mediated rejection. In both cases, corticosteroid therapy was progressively tapered to a maintenance dose of 5 mg/day following the diagnosis of CSCR. Conversely, the third patient developed CSCR while receiving a maintenance prednisone dose of 20 mg/day, emphasizing that the condition may occur not only after pulse corticosteroid therapy but also during prolonged exposure to moderate maintenance doses. This observation is consistent with previous reports demonstrating that CSCR may develop across a broad range of corticosteroid dosages and exposure durations [6].

From a practical management perspective, treatment should be individualized according to the severity of visual symptoms, retinal involvement, and the patient’s immunological risk profile. Recipients with recent transplantation, previous rejection episodes, or high immunological risk require particularly cautious corticosteroid tapering and close nephrological follow-up. Throughout follow-up, our patients underwent multidisciplinary monitoring, including serial assessment of serum creatinine, proteinuria, donor-specific antibodies when indicated, and regular ophthalmologic evaluations. Corticosteroid reduction was not associated with acute rejection episodes or deterioration of graft function in the first two patients. In contrast, the third patient had established chronic allograft dysfunction before the diagnosis of CSCR and continued to exhibit persistent renal impairment during follow-up. These findings support the feasibility of cautious corticosteroid tapering in selected kidney transplant recipients with CSCR when performed within a multidisciplinary framework balancing ophthalmologic benefits against immunological risk.

When corticosteroid reduction is not feasible or when CSCR persists despite optimization of immunosuppressive therapy, alternative ophthalmologic interventions may be considered.

Current evidence supports half-dose photodynamic therapy (PDT) as one of the most effective treatments for chronic or persistent CSCR [11]. Other options include focal laser photocoagulation for extrafoveal leakage points and subthreshold micropulse laser therapy [11]. Anti-vascular endothelial growth factor (anti-VEGF) therapy may be useful in selected cases complicated by secondary choroidal neovascularization but is not routinely recommended for uncomplicated CSCR [10].

In addition, factors such as disease severity, recurrence, and the presence of systemic comorbidities, including hypertension, post-transplant diabetes mellitus, and overweight, may influence visual prognosis and should be considered in therapeutic decision-making [3,4,11,12].

The clinical course of CSCR is generally favorable following corticosteroid reduction while maintaining adequate immunosuppression. Previous studies suggest that a 25% to 50% decrease in corticosteroid dose may accelerate symptom resolution, with an average recovery time of approximately two months, compared with longer delays when corticosteroid doses are maintained. In our series, all patients achieved complete visual recovery, with resolution occurring within weeks to a few months, in agreement with published data [5,7,8,11].

Case 3 highlights a distinct presentation of post-transplant CSCR, characterized by incidental diagnosis during routine ophthalmologic screening in an asymptomatic patient. This silent presentation, also reported in patients receiving long-term corticosteroid therapy [3,8], underscores the risk of underdiagnosis and further supports the need for systematic and prolonged ophthalmologic follow-up in kidney transplant recipients, particularly those exposed to repeated corticosteroid courses.

Finally, several studies have demonstrated the impact of CSCR on patients’ quality of life, even after recovery of visual acuity, due to persistent visual disturbances such as reduced contrast sensitivity, metamorphopsia, and color vision abnormalities [2,12]. A multidisciplinary approach involving close collaboration between nephrologists and ophthalmologists is therefore essential to optimize patient management, ensuring both graft survival and preservation of visual function [5,11,12].

## Conclusions

CSCR is a clinically relevant ophthalmologic complication in kidney transplant recipients. The occurrence of both symptomatic and asymptomatic presentations underscores the importance of systematic ophthalmologic monitoring, particularly in patients exposed to prolonged or repeated corticosteroid therapy. Early recognition and a multidisciplinary management approach, including individualized corticosteroid tapering when feasible, may improve visual outcomes while preserving graft function.
